# Acute Exacerbation of Rheumatoid Arthritis-Associated Interstitial Lung Disease After Thoracic Surgery in the Absence of Usual Interstitial Pneumonia Pattern on CT

**DOI:** 10.7759/cureus.41155

**Published:** 2023-06-29

**Authors:** Tatsuya Kida, Yutaka Usuda, Teisei Kobashi, Masakazu Sumitomo

**Affiliations:** 1 Anesthesiology, Yokosuka Kyōsai Hospital, Yokosuka, JPN; 2 Intensive Care Unit, Yokosuka Kyōsai Hospital, Yokosuka, JPN

**Keywords:** lung surgery, usual interstitial pneumonia pattern, acute exacerbation, rheumatoid arthritis, interstitial lung disease

## Abstract

The usual interstitial pneumonia (UIP) pattern observed on chest computed tomography (CT) is considered a risk factor for the development of postoperative acute exacerbation in interstitial lung disease (ILD). However, the risk factors for acute exacerbation in patients with rheumatoid arthritis (RA)-associated ILD have not been adequately investigated. We present a case of postoperative acute exacerbation after thoracic surgery in a 73-year-old man with RA-ILD and non-UIP pattern on chest CT. This case report emphasizes that postoperative acute exacerbation can develop even in the absence of a radiological UIP pattern.

## Introduction

Patients with interstitial lung disease (ILD) are at risk of developing acute exacerbation after thoracic surgery, and the mortality rate after acute exacerbation is reported to be 43.9% [1,2]. ILD is a common manifestation of rheumatoid arthritis (RA) and contributes to mortality in patients with RA. Patients with RA may develop lung cancer and potentially require thoracic surgery [3]. The usual interstitial pneumonia (UIP) pattern on computed tomography (CT) is a risk factor for postoperative acute exacerbation of ILD. However, it is unclear whether the risk factor can be applied to patients with RA-associated ILD (RA-ILD) because previous reports are mainly limited to patients with interstitial pulmonary fibrosis (IPF) [1,4,5]. Therefore, the specific risk factors for postoperative acute exacerbation of RA-ILD after thoracic surgery have not been adequately investigated, and unanticipated deaths still occur from postoperative acute exacerbation. Here, we describe a case of postoperative acute exacerbation after thoracic surgery in a patient with RA-ILD and a non-UIP pattern on chest CT.

## Case presentation

**Figure 1:**
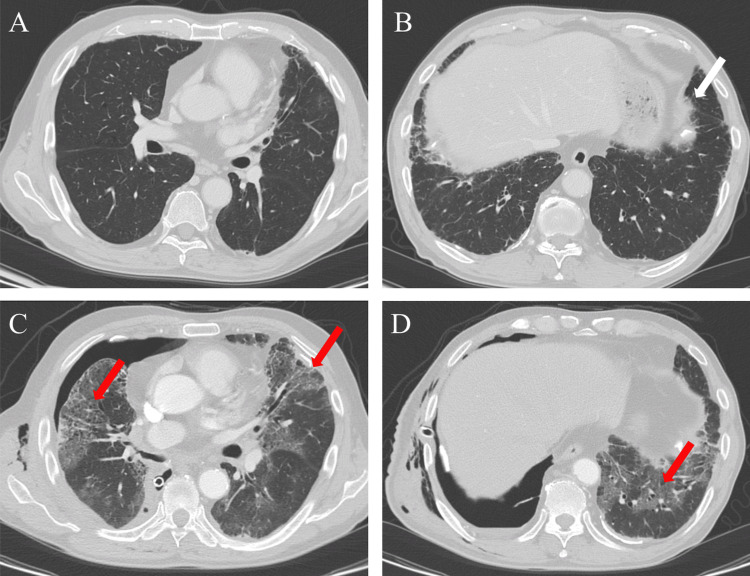
Perioperative high-resolution computed tomography images. (A, B) Preoperative computed tomography image demonstrating indeterminate for usual interstitial pneumonia pattern. White arrows showing lower-lobe subtle reticular abnormality without honeycombing in the subpleural areas with a basal predominance. (C, D) Chest computed tomography scan taken four days after lung surgery. Red arrows showing widespread diffuse bilateral ground-glass opacity with bronchial dilatation.

A 73-year-old man (height, 163 cm; weight, 64 kg; American Society of Anesthesiologists class II) was admitted to our hospital for evaluation of lung lesions. Due to a history of asbestos exposure, the patient had undergone routine chest CT over the past seven years, which showed subpleural non-fibrotic interstitial lung abnormalities with no remarkable changes. He was diagnosed with RA four years prior and treated with oral prednisolone (2 mg/day) and methotrexate (8 mg/week). He was a smoker (Brinkman index: 880) and had a history of chronic obstructive pulmonary disease. He had no history of an acute exacerbation of ILD and did not complain of dyspnea or cough. Chest CT revealed nodular shadows in the middle and lower right lobes (S4 and S9), and CT-guided percutaneous needle biopsy revealed combined large-cell neuroendocrine carcinoma with squamous cell carcinoma. The patient received four cycles of carboplatin and etoposide, which resulted in a partial response. He was scheduled for right lower and middle lobectomy and lymph node dissection for lung cancer as salvage surgery. Preoperative high-resolution chest CT showed bilateral subtle subpleural reticulations without honeycombing, which was diagnosed as indeterminate for UIP pattern (Figure 1A, 1B). The laboratory findings were as follows: KL-6, 917 U/mL; lactate dehydrogenase, 337 U/L; C-reactive protein, 1.82 mg/dL; and hemoglobin, 9.9 g/dL. The pulmonary function test showed no respiratory dysfunction; forced expiratory volume (FEV) in one second was 2.48 L, percent predicted FEV in one second was 76.1%, vital capacity (VC) was 3.19 L, and percent predicted VC was 93.8%. Transthoracic echocardiography revealed normal left ventricular function, with no evidence of elevated right ventricular pressure.

On arrival in the operating room, his initial oxygen saturation measured by pulse oximetry (SpO_2_) was 95% in room air. After performing epidural anesthesia at the T5-6 intravertebral level, the patient was anesthetized using propofol and fentanyl, and rocuronium was used as a muscle relaxant. The trachea was intubated with a left-sided 37 Fr double-lumen tube (Broncho-Cath®, Mallinckrodt Medical Inc., Athlone, Ireland). Anesthesia was maintained using sevoflurane and remifentanil. After initiating one-lung ventilation, the dependent lung was ventilated using pressure-control ventilation, with peak inspiratory pressure maintained below 25 cm H_2_O and a positive end-expiratory pressure of 3-5 cm H_2_O. The fraction of inspired oxygen was increased to 1.0 for a total of 50 minutes to maintain the SpO_2_ above 88%. Continuous positive airway pressure was not applied to the affected lung. The patient underwent right lower and middle lobectomy and lymph node dissection. The operative time was 356 minutes. The patient’s trachea was extubated in the operating room and was transferred to the intensive care unit. Postoperative pathological examination of the excised specimen revealed a pathological UIP pattern and small cell carcinoma because of the morphological findings. Asbestos bodies were not observed in any of the specimens. Based on the pathological and preoperative radiological findings and the patient’s history of RA, the cause of ILD was determined to be associated with RA.

The patient's right lung collapsed with an air leak, and he received oxygen via a nasal cannula at 1-4 L/minute for the first three days, postoperatively. Four days after thoracic surgery, his oxygenation worsened. Chest CT revealed widespread, diffuse bilateral ground-glass opacities with bronchial dilatation (Figures 1C, 1D). Because there was no evidence of heart failure or pulmonary embolism, the patient was diagnosed with postoperative acute exacerbation of RA-ILD. After tracheal intubation, the patient received steroid and cyclophosphamide pulse therapy. The pulmonary fistula of the right lung was not closed, and respiratory failure progressed despite interventions. The patient died of respiratory failure 35 days after the surgery.

## Discussion

ILD is a group of respiratory diseases characterized by fibrosis of the lungs, with a prevalence ranging from 6.3 to 71 per 100,000 people [6]. Possible causes of ILD include autoimmune diseases such as RA, asbestos or drug exposure, and idiopathic diseases such as IPF. To help the diagnosis of IPF in patients with ILD, four diagnostic categories of CT patterns have been defined: UIP, probable UIP, indeterminate for UIP, and alternative diagnosis [7]. In non-perioperative cases, the UIP pattern on chest CT was reported as a risk factor for developing an acute exacerbation of RA-ILD [8,9]. Therefore, a radiological UIP pattern is a potential risk factor for postoperative acute exacerbation of RA-ILD. Our patient developed postoperative acute exacerbation despite the preoperative chest CT revealing an indeterminate UIP pattern. The cause of the acute exacerbation may be a postoperative diagnosis of pathological UIP pattern. Assegai et.al. revealed that the sensitivity of a radiological to a pathological UIP pattern was 45% [10]. Their report implies that a pathological UIP pattern cannot be ruled out even if a UIP pattern is not shown on a preoperative chest CT. Patients with RA-ILD with a pathological UIP pattern have a worse prognosis than those without such a pattern [11]. Considering that the postoperative diagnosis was a pathological UIP pattern, our patient was probably susceptible to developing postoperative acute exacerbation.

Among the known risk factors for developing postoperative acute exacerbation, our patient had the following: male sex, preoperative steroid use, lobectomy, and long operation time. Although both IPF and RA-ILD cause pulmonary fibrosis and show similar radiological findings on chest CT, it is unclear whether these risk factors are applicable to patients with RA-ILD, owing to their different pathogenetic features. For example, methotrexate usage may be a potential risk factor for postoperative acute exacerbation of RA-ILD because it was shown to be a risk factor in non-perioperative cases [9]. Even if multiple risk factors are preoperatively recognized, it is often difficult to predict the likelihood of postoperative acute exacerbation in individual cases. In other words, it is always a clinical dilemma whether to stop the surgery or how the surgical procedure should be planned. The ILD-gender-age-physiology (ILD-GAP) model was developed to predict the prognosis of patients with ILD and is useful in predicting the prognosis after lung surgery [12,13]. However, this model has a disadvantage in that the ILD-GAP index cannot be calculated in cases where the diffusion capacity for carbon monoxide cannot be measured, such as in our hospital. The Assess Respiratory Risk in Surgical Patients in Catalonia (ARISCAT) score was developed to predict the risk of postoperative pulmonary complications [14]. Hosoki et.al. revealed that the ARISCAT score of ≥45 was a risk factor for postoperative acute exacerbation of ILD [15]. Notably, their study participants included not only IPF patients but also RA-ILD patients. Our patient had a preoperative ARISCAT score of 69 (high risk of postoperative pulmonary complications) (Table 1).

**Table 1:** ARISCAT Score. Asterisks indicate the scores in our case. The risk of postoperative pulmonary complication is as follows: Low risk ( < 26 points), Intermediate risk (26–44 points), and High risk (≧45 points). ARISCAT, Assess Respiratory Risk in Surgical Patients in Catalonia.

ARISCAT score component	Score
Age, years	
≦50	0
51-80	3*
> 80	16
Preoperative SpO_2_, %	
≧96	0
91-95	8*
≦90	24
Respiratory infection in the last month	17
Preoperative anemia (≦10 g/dl)	11*
Surgical incision	
Peripheral	0
Upper abdominal	15
Intrathoracic	24*
Duration of surgery, hours	
≦2	0
> 2 to 3	16
>3	23*
Emergency procedure	8
The total score of our patient	69

The ARISCAT score includes various risk factors such as operation time and surgical incision site, which may be helpful in predicting postoperative acute exacerbation. It is important to note that all cases with intra-thoracic surgery of more than three hours have a score of ≥47 points. Therefore, in cases with an ARISCAT score of ≥45, if possible, surgical approaches to reducing operative time may be considered. In our case, the nodular shadows in the middle and lower lobes could not be ruled out as lung cancer, and both lobes had to be removed, resulting in a prolonged operation time. Given that the postoperative diagnosis was small cell carcinoma, the lung resection could have been aborted if an intraoperative pathological examination had been performed, which may have led to the avoidance of acute exacerbation. Further studies are required to develop a practical scoring system for patients with RA-ILD to predict the likelihood of postoperative acute exacerbation with greater accuracy or to validate the ARISCAT score, including reconsideration of the cutoff value.

## Conclusions

We reported a case of postoperative acute exacerbation after thoracic surgery in a patient with RA-ILD without a UIP pattern on chest CT. The patient, unfortunately, died due to respiratory failure 35 days after surgery. The present case suggests that clinicians need to manage the perioperative course taking into consideration the development of postoperative acute exacerbation in patients with RA-ILD with high ARISCAT scores even if preoperative chest CT shows no UIP pattern. In other words, patients with high ARISCAT scores should consider the option of postponing surgery. Identifying modifiable risk factors for postoperative acute exacerbation is paramount and a research topic for future studies.
